# Response of FcεRI‐bearing leucocytes to omalizumab in chronic spontaneous urticaria

**DOI:** 10.1111/cea.13566

**Published:** 2020-02-07

**Authors:** Mehran Alizadeh Aghdam, Edward F. Knol, Mignon van den Elzen, Constance den Hartog Jager, Harmieke van Os‐Medendorp, André C. Knulst, Henny G. Otten, Heike Röckmann

**Affiliations:** ^1^ Division Internal Medicine and Dermatology Department Dermatology/Allergology University Medical Center Utrecht Utrecht The Netherlands; ^2^ Division of Laboratories, Pharmacy and Biomedical Genetics Center of Translational Immunology University Medical Center Utrecht Utrecht The Netherlands

**Keywords:** basophil degranulation, FcεRI, high‐affinity IgE receptor, omalizumab, skin, therapy response, urticaria

## Abstract

**Background:**

The pathogenesis of chronic spontaneous urticaria (CSU) and the mechanism of action of omalizumab in CSU remain unclear.

**Objective:**

In this study, we assessed the responsiveness and FcεRI expression of various subsets of leucocytes in patients with CSU treated with omalizumab.

**Methods:**

In this prospective cohort study, 30 patients were treated with 6 administrations of 300 mg omalizumab every 4 weeks, followed by a follow‐up period of 12 weeks. FcεRI expression and the percentage of basophils, monocytes, and dendritic cell subsets were analysed before and during treatment, and after follow‐up. In addition, anti‐IgE– and C5a‐induced basophil degranulation was measured. The results were correlated with disease activity and response to omalizumab.

**Results:**

In addition to a rapid and significant reduction in FcεRI on basophils, we demonstrated a reduction in FcεRI on plasmacytoid dendritic cells during omalizumab treatment, which persisted until 3 months after discontinuation. FcεRI expression on basophils and its reduction did not correlate with the treatment response. Omalizumab led to an increased percentage of basophils in blood but not of the other FcεRI‐bearing leucocytes. Basophil responsiveness was differentially affected; anti‐IgE–, but not C5a‐induced basophil degranulation increased during the treatment. Apart from clinical non‐responders showing a stronger increase in anti‐IgE–induced basophil degranulation over a period time, no differences were found in omalizumab responders vs non‐responders.

**Conclusions/Clinical Relevance:**

FcεRI expression on basophils decreased rapidly, while anti‐IgE–induced degranulation significantly increased due to omalizumab treatment in patients with CSU, persisting at least for 3 months after stopping the treatment. None of the markers were able to predict the effectiveness of treatment. Whether basophils play a role in omalizumab responsiveness in CSU remains unclear.

**Abbreviations**
CSU: chronic spontaneous urticaria
mDC: myeloid dendritic cells
pDC: plasmacytoid dendritic cells
PRO: patient‐reported outcomes
UAS7: Urticaria Activity Score during 7 days

## 1 INTRODUCTION

Chronic spontaneous urticaria (CSU) manifests as a skin disease with a sudden onset of weals, which last longer than 6 weeks. The disease duration ranges from 1 to 5 years or even longer in more severe cases.^1^ Omalizumab is effective as a third‐line treatment in a majority of CSU patients with insufficient response to a fourfold dose of antihistamines.^2^, ^3^ It is administered subcutaneously and reaches peak serum concentrations after an average of 7‐8 days. Clearance of the monoclonal antibody is slow, with a terminal half‐life of 19‐22 days,^4^ which allows for relatively long treatment intervals of 4 weeks. A rapid clinical response can be seen in a proportion of the patients after the first omalizumab dose administration; however, other patients require multiple doses to reach a well‐controlled disease status.^5^

Omalizumab is a humanized monoclonal antibody, which binds to the Cε3 domain of free IgE, thereby preventing it from binding to Fc epsilon RI (FcεRI).^6^ Depletion of free IgE by omalizumab leads to a down‐regulation of the FcεRI on mast cells in a majority of patients.^7^, ^8^ Mast cells are considered to be the most important effector cells in CSU. In addition, a role for basophils has been suggested in certain urticaria phenotypes.^9^ Basophil numbers are inversely related to urticaria severity.^10^, ^11^ An increased presence of basophils in the skin and decreased numbers in peripheral blood suggest that basophils are recruited to the affected skin sites.^12^, ^13^, ^14^

Recent studies investigating the response of skin mast cells to omalizumab in allergic patients showed down‐regulation of FcεRI expression after 1‐2 months.^15^, ^16^, ^17^ Therefore, other cell types, such as basophils and dendritic cells, might account for the rapid clinical effect of omalizumab.^18^, ^19^

Decreased degranulation of basophils after stimulation via FcεRI was demonstrated in patients with urticaria compared to that in healthy controls.^10^ It is not known if responses to other stimuli, such as C5a (which activates basophils via a G‐protein–coupled pathway), are affected.^20^ Besides basophils and mast cells, other myeloid cells can also express FcεRI on their surfaces.^21^ The presence of FcεRI has been demonstrated on monocytes and different types of dendritic cells, more profoundly in patients with allergies than in healthy individuals. In allergic rhinitis patients, expression of FcεRI on the different myeloid cells depended on serum IgE concentration, and treatment with omalizumab reduced the expression of FcεRI on basophils, mast cells, and DCs.^22^, ^23^, ^24^ Recently, Deza et al suggested that the baseline expression of basophil FcεRI was a potential immunological predictor of responsiveness to omalizumab in urticaria. Furthermore, they found that patients who responded to omalizumab treatment had a rapid reduction in the levels of basophil FcεRI during treatment. Given the potential role of FcεRI‐bearing leucocytes, in particular basophils, in the pathogenesis and/or treatment response to omalizumab, we evaluated the role of FcεRI‐bearing cells in CSU patients treated with omalizumab.

## 2 METHODS

### 2.1 Design and population

This monocentric exploratory prospective cohort study was performed in the University Medical Center Utrecht, the Netherlands, from 2015 until 2017. We included 30 patients according to the following criteria: age ≥ 18 years, active diagnosis of CSU (weekly urticaria activity score [UAS7]) ≥ 16, in‐clinic UAS ≥ 4 on the day of the first omalizumab administration, and insufficient response to a four‐times daily administration of antihistamines. Exclusion criteria were clearly defined underlying aetiology for chronic urticaria (eg chronic inducible urticaria [CINDU]), a history of malignancy, known hypersensitivity to omalizumab, and pregnancy. Routine administration of immunosuppressants, including prednisolone and cyclosporine A (CsA) was discontinued with washout periods of 3 months prior to treatment with omalizumab. If prednisolone was used as a rescue medication, a washout period of 2 weeks was maintained.

After a screening period of up to 2 weeks, eligible patients received six doses of 300 mg omalizumab every 4 weeks. After the last omalizumab administration, the patients were observed during a follow‐up period of 3 months. The patients were kept on a treatment with fourfold dose of H1 antihistamines throughout the study period. As a rescue medication, patients were allowed to use prednisolone, up to 30 mg daily. All other CSU‐related medications were discontinued.

Disease activity was measured throughout the study using the UAS7.^25^ Treatment response is defined as a UAS ≤ 6 at week 24 of treatment. Improvement by a minimal important difference (MID) is defined as a reduction in 10 UAS7 points.^26^ All the patients provided written informed consent, and the study was approved by the local ethics committee (protocol number 15‐167).

### 2.2 Blood collection

Blood samples were collected at the following time‐points: at baseline (T0) and at different time‐points after the first injection: 6 hours (T0.25), 1 day (T1), 1 week (T7), 2 weeks (T14), 1 month (second dose, T28), 1 month and 2 hours (T28.08), 2 months (T56), 3 months (T84), 4 months (T112), 5 months (T140) and 8 months (follow‐up, T224). EDTA blood and gel‐separated serum were placed on ice immediately after venipuncture. Blood was also collected from nine self‐reported healthy controls for baseline analysis. All serum samples were allowed to coagulate for 60 minutes. Serum and plasma were obtained by centrifugation and stored at −80°C. Total IgE was determined using the ImmunoCap assay according the manufacturer's instructions (Thermo Fisher Scientific).

### 2.3 Leucocyte subset determination

Leucocytes subsets were identified using an antibody panel containing CD45‐PO (Life Technologies) for lymphocytes; CD123‐PerCPCy5.5 (BD Pharmingen), CD203c APC (Sony), HLA‐DR‐PB (Sony), and CD41‐PE‐Cy7 for basophils; and CD45‐PO (Life Technologies) and CD14‐APC‐H7 (BD Pharmingen) for monocytes. To distinguish between the three different subsets of dendritic cells (DCs), an antibody panel containing HLA‐DR‐PE‐CY7 (BioLegend), CD11c‐PB (BioLegend), and CD123 PerCP Cy5.5 (BD Pharmingen) was used for plasmacytoid dendritic cells (pDCs). For two subsets of myeloid dendritic cells (mDCs), CD14‐V500 (BD), HLA‐DR‐PE‐CY7 (BioLegend), CD1c‐APC‐Cy7 (BioLegend), and CD141‐APC (Miltenyi) were used. Leucocyte subset quantities were depicted as the percentage of cells within the total leucocyte measures/numbers.

### 2.4 Quantification of FcεRI expression

Whole blood samples were divided into aliquots of 75 µL each to carry out staining of basophils, monocytes, dendritic cells (DC), or an isotype control. All the cells were stained for either FcεRI (CRA‐1, eBioscience) or an IgG2b isotype control (Sony) for 30 minutes at 4°C in the dark. Following washing, cells and QIFIKIT beads (Dako, Glostrup, Denmark) were simultaneously stained with a saturated solution of goat anti‐mouse IgG FITC to determine the absolute FcεRI expression, quantified as antibody binding capacity (ABC). The QUIFIKIT contains five bead populations with a distinct and known amount of monoclonal mouse antibody bound per microsphere bead. By constructing a calibration curve based on the fluorescence intensity of different populations plotted against their known antibody density, FcεRI expression on different cell types can be interpolated based on their mean fluorescence intensity (MFI). The specific antibody binding capacity (SABC) is then calculated by subtracting the calculated ABC for corresponding isotype controls from the anti‐FceRI ABC.

### 2.5 Basophil activation test

Heparin anti‐coagulated blood samples were stimulated for 30 minutes at 37°C with increasing concentrations of anti‐IgE (0.03, 0.1, 0.3, and 1 µg/mL) (Vector laboratories) or C5a (83 and 200 ng/mL) (R&D Systems) in RPMI‐1640 medium (Gibco, Life Technologies) containing 1 ng/mL IL3 (R&D Systems). Leucocytes were stained with an antibody cocktail of CD45‐PO (Life Technologies), CD123‐FITC (BioLegend), HLA‐DR‐PB (Sony), CD63‐PE (Monosan), CD41 PE‐CY7 (Beckman Coulter), or CD203c‐APC (Sony). Basophils were defined as CD45^+^ CD203c^+^ CD123^+^ and HLA‐DR^‐^ CD41^‐^. Basophil degranulation was quantified by determining the percentage of CD63‐binding basophils. The threshold for basophil degranulation was set between degranulated and resting basophils.

### 2.6 Statistical analysis

Differences in cell counts, basophil activation test (BAT) results, and FcεRI density in time were analysed using Wilcoxon matched‐pairs signed‐rank tests. Analyses between different responder groups were performed using Mann‐Whitney *U* tests. Correlation analysis was performed using Spearman's rank correlation or Pearson's correlation if appropriate. Regarding the UAS7 score, the difference between each time‐point and baseline was tested using Wilcoxon matched‐pairs signed‐rank tests. Statistical analysis was performed using IBM SPSS Statistics version 21 or GraphPad Prism version 7.02. Graphs were plotted using Microsoft Visio 2010 or GraphPad Prism version 7.02.

## 3 RESULTS

### 3.1 Clinical efficacy of omalizumab

Thirty patients (median age 42 years [range of 21‐700; 73% female]) with a median UAS7 score at baseline of 31.5 points were enrolled in the study. Patient characteristics (Table S1) corresponded with the CSU population in our clinic and current studies in literature.^27^

**Figure 1:**
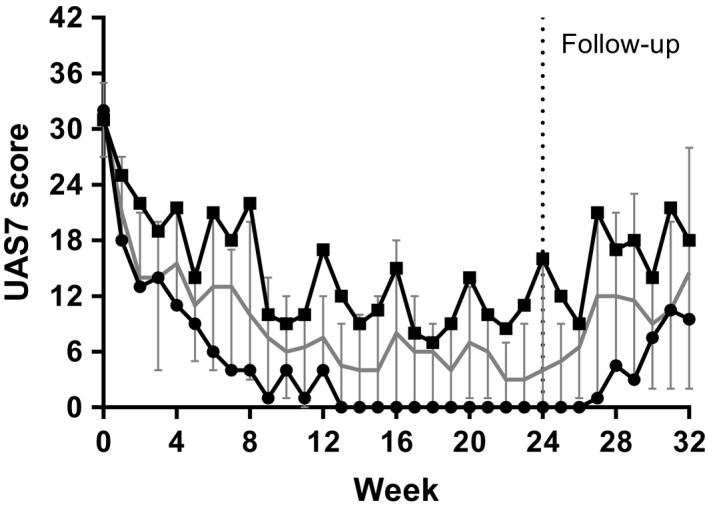
Median values of UAS7 for responders and non‐responders improve during omalizumab treatment. Median values of UAS7 at baseline, during omalizumab treatment, and during follow‐up are presented for responders and non‐responders. At the start of week 20, the final dose of omalizumab was administered, which initiated the follow‐up period after week 24 (dotted line). Subjects who restarted omalizumab during the follow‐up period were excluded from data analysis. 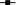 Subjects with UAS7 > 6 at week 24; n = 15 non‐responders/partial responders; 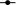 Subjects with UAS7 ≤ 6 at week 24; n = 15 responders; 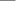 Overall median + confidence interval

Figure 1 shows the weekly median values of UAS7; the patients were differentiated into omalizumab responders and non‐responders. Fifteen patients (50%) showed a UAS7 score of six or lower (median 0) at 4 weeks after the last omalizumab administration (24 weeks) and were defined as responders. Fourteen patients showed a UAS7 score of seven or higher (median 16) at week 24 and were defined as non‐responders. The UAS7 score of one patient was missing at week 24 and was marked as non‐responder based on the last known UAS7 score.

Improvement by a minimal important difference (MID) of 10 UAS7 points at week 24 was observed in 23 patients (76.6%), which included nine complete responders (UAS7 = 0). Due to worsening of the disease, 11 patients, of which 6 (55%) were presented as responders, restarted omalizumab treatment during follow‐up. Subjects who restarted omalizumab during the follow‐up period were excluded from the follow‐up data analysis. In absolute numbers, the number of patients who were excluded was 1 in week 25, 2 in week 26, 3 in week 28, 4 in week 29, 9 in week 30, and 11 in week 32.

### 3.2 FcεRI expression on basophils, pDCs and mDC CD1cs decreases during treatment

**Figure 2:**
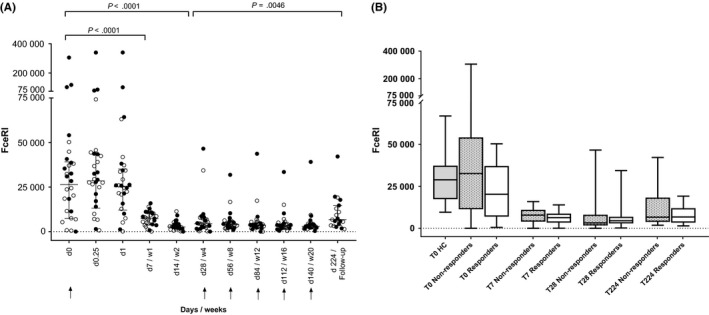
Median FcεRI expression on basophils decreases during omalizumab treatment. A, FcεRI expression on basophils at various time‐points. (Responder: ○, Non‐responder: ●)_Arrows indicate omalizumab administration. Counts expressed as a median of molecules per basophil. B, FcεRI expression on basophils at selected time‐points in healthy controls (HC) (grey box‐plot), non‐responders (dotted box‐plot), and responders (white box‐plot)

In peripheral blood, we determined FcεRI expression on basophils, monocytes, pDCs, and two subsets of mDCs (mDC CD141 and mDC CD1c) at specific time‐points before, during, and after treatment. A large and significant difference in FcεRI expression on basophils was found at T7 (*P* < .0001) and all other time‐points, including after 3‐month follow‐up (T224) compared to that at baseline (T0) (Figure 2). Reduction in FcεRI expression did not differ significantly between responder and non‐responder groups. The decline in FcεRI expression showed a weak correlation with the decline in UAS7 score, 1 week after baseline (*r* = .675, *P* = .008). A similar decline in FcεRI expression after omalizumab treatment was observed for pDC and mDC CD1c (data not shown).

Furthermore, FcεRI expression on basophils at baseline did not differ between omalizumab responders and non‐responders (*P* = .202), healthy controls and responders (*P* = .215), and healthy controls and non‐responders (*P* = .682) (Figure 2A and B). Moreover, we did not find a statistical difference (*P* = .408) when comparing extremes response groups: complete responders (n = 9, all UAS7 = 0) to extremely poor responders (n = 7, UAS > 16; median UAS7 = 25).

Total IgE (available for 28 of the 30 patients) did not differ significantly either between responders (n = 15, median: 170.0 kU/L) and non‐responders (n = 13, median 81.9 kU/L; *P* = .387) or between patients with self‐reported atopy (n = 15, median: 122.0) and without atopy (n = 13, median: 107.0, *P* = .467). In addition, no correlation was found between total IgE levels and baseline basophil FcεRI expression (*r* = .081, *P* = .682). However, when removing one extreme outlier with a total IgE > 5000 kU/L, a moderate correlation between baseline total IgE and baseline basophil FcεRI expression (*r* = .4, *P* = .037) was seen, which was comparable to that mentioned in a recent study.^28^

### 3.3 Only basophils percentages increase, other FcεRI‐bearing leucocytes remain stable

**Figure 3:**
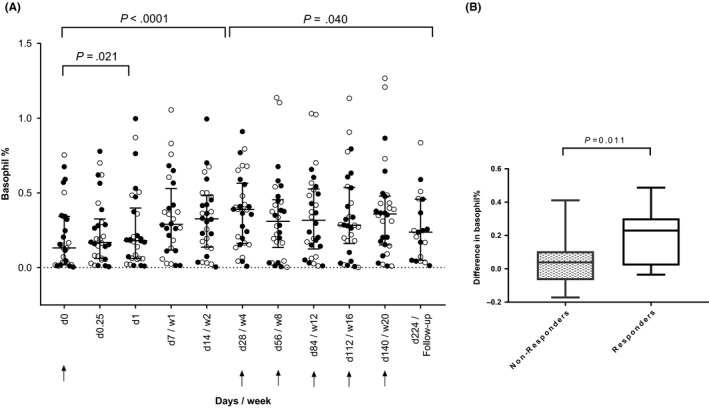
Percentage of basophils measured in a patient's sample increase during omalizumab treatment. A, Portion of basophils measured at various time‐points. (Responder: ○, Non‐responder: ●). Arrows indicate omalizumab administration. B, Basophil percentage after 1 wk of omalizumab treatment compared to that at baseline in omalizumab non‐responders (dotted box‐plot) and responders (white box‐plot) at week 24

We measured percentages of basophils, monocytes, pDCs, mDCs (CD141) and mDCs (CD1c) at baseline (T0), several time‐points during omalizumab treatment, and at 3‐months follow‐up (T224). Median percentage of basophils measured in blood showed an increase during omalizumab treatment (Figure 3) within 1 day (median: 0.18) compared with baseline (median: 0.13), reaching a maximum at 4 weeks. At follow‐up, the median percentage of basophils (median: 0.24) was still higher compared with that of baseline. Healthy controls showed a higher percentage of basophils compared with CSU patients at baseline (*P* < .001). There was no difference in median percentage of basophils between non‐responders and responders at any given time‐point. However, we did notice a significantly faster increase in the percentage of basophils 1 week after the first omalizumab administration in the responder group compared with that of the non‐responder group (*P* = .011, [Figure 3B]).

There was no significant change in median percentages of the other analysed leucocytes, such as eosinophils, monocytes, pDCs, mDC CDC141^+^, and mDC CD1c^+^ after omalizumab treatment (data not shown).

### 3.4 Anti‐IgE– but not C5a‐induced basophil degranulation increases during omalizumab treatment

**Figure 4:**
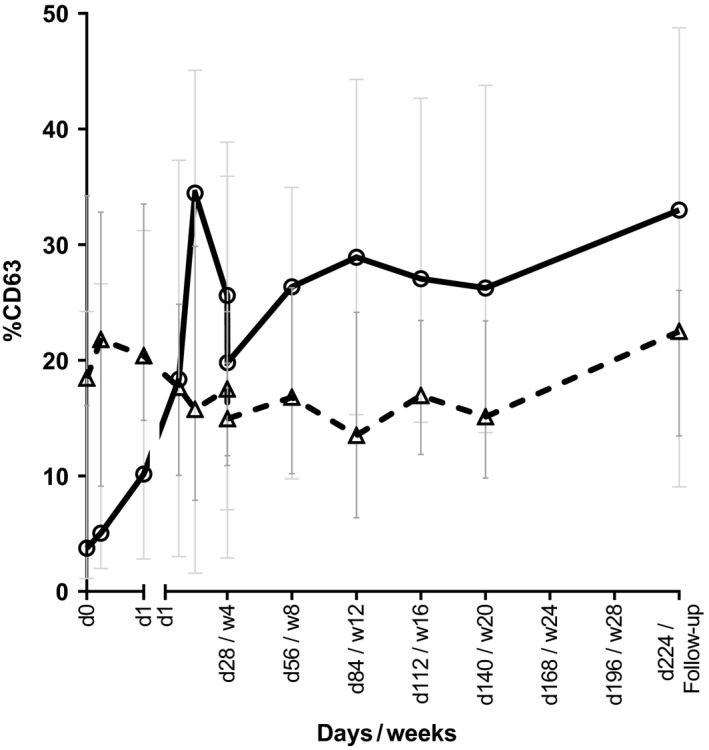
Anti‐IgE‐induced basophil activation (1 µg/mL) increases and C5a‐induced basophil activation (200 ng/mL) decreases during omalizumab treatment. Basophils were stimulated with 1 µg/mL anti‐IgE (‐Ο‐) or 200 ng/mL C5a (‐Δ‐) for 30 min, and activation was determined as % cells with increased CD63 expression by flow cytometry

A significant increase in anti‐IgE–induced (1 µg/mL) basophil activation was observed after 24 hours (*P* = .042), which was maintained for all the subsequent time‐points (Figure 4). A similar pattern was seen after stimulation with suboptimal concentrations of anti‐IgE (0.3, 0.1, and 0.03 µg/mL). A significant difference was not observed in baseline anti‐IgE–induced basophil degranulation between responders and non‐responders (*P* = .148). However, non‐responders showed a significantly stronger increase in anti‐IgE–induced basophil activation at T28 compared with T0 (*P* = .003) and at T224 compared with T28 (*P* = .049), while responders did not show a significant difference (*P* = .104 and *P* = .742, respectively) (Figure S1).

Contrary to the anti‐IgE–induced FcεRI‐mediated basophil degranulation, C5a‐induced degranulation showed a significant reduction (Figure 4) after 1 month of treatment (*P* = .025) and at all subsequent time‐points during treatment. This reduction in C5a‐induced basophil degranulation returned to baseline levels during the follow‐up period. Responders and non‐responders showed a similar pattern of C5a‐induced basophil degranulation. When basophils were stimulated with a suboptimal concentration of 83 ng/mL, a similar effect was observed as that with stimulation with an optimal concentration of C5a of 200 ng/mL.

## 4 DISCUSSION

In this study, the effect of omalizumab treatment in CSU on different FcεRI‐bearing leucocytes was investigated.

A decline of FcεRI on basophils was observed within 1 week after initiation and during omalizumab treatment, as shown in earlier studies.^7^, ^8^ In this study, we present several important and novel findings. The decreased FcεRI expression on basophils persisted up to 12 weeks after the last dose and was also detected on the mDC CD1c subset and pDCs. However, this decrease was not found on monocytes or on the mDC CDD141 subset. We also observed that the basophil responsiveness was differentially affected by omalizumab treatment, since anti‐IgE–, but not C5a‐induced basophil degranulation increased during the treatment.

Another important finding was that we could not relate either the baseline expression or the decline of FcεRI expression on basophils to the clinical effect of omalizumab. A recent study^29^ suggested that baseline FcεRI expression could predict omalizumab treatment response. In a study of Deza et al, non‐responders showed significantly lower baseline levels of FcεRI expression compared with healthy controls and responders. Furthermore, a decrease in FcεRI expression was mainly observed in responders. In our study, we found a large overlap in the FcεRI levels between healthy controls, responders, and non‐responders, and no statistical difference was found among the three groups. Moreover, we did not find a statistical difference (*P* = .408), when complete responders (n = 9, median UAS7 = 0) were compared to extreme poor responders (n = 7, UAS > 16, median: 25). Moreover, the change in FcεRI levels did not correlate with the change in urticaria activity (UAS7 scores) per patient. A possible explanation for the discrepancy between the two study results might be a difference in patient population. Although demographics between the two studies were fairly similar, patient‐reported outcomes differed noticeably, since 81% of the patients in the study by Deza et al achieved a UAS7 score of ≤6 or a ≥90% reduction in UAS7 vs only 50% in our study.

The decreased FcεRI expression levels persisted in patients for at least 3 months even after discontinuing the omalizumab treatment. In a similar study, Jörg et al^30^ found that FcεRI expression on basophils was decreased during omalizumab treatment and up to 2 months after the last dose. These results suggest lasting effects of omalizumab, which might explain why a proportion of the patients show a beneficial effect even after long intervals.^31^

The median percentage of peripheral blood basophils showed a rapid and significant increase over time (Figure 3), which has been described previously during omalizumab and steroid treatment.^7^, ^11^, ^32^ None of the other leucocytes showed such a reaction to omalizumab treatment, which may support a prominent role of basophils. There was no difference between omalizumab responders and non‐responders. However, a significantly stronger increase in the percentage of basophils was observed in responders compared with non‐responders solely 1 week after the first administration of omalizumab.

These findings point towards a possible compartmental shift, in which basophils remain in the circulating blood rather than migrate to the affected skin, as suggested by Grattan et al^11^ In our study, 4 of the 30 patients (1 responder, 3 non‐responders) reported prednisolone use at some time‐points during the study period, which might have influenced the blood basophil numbers.

An interesting new finding was that the degranulation of basophils was significantly increased after the cross‐linking of FcεRI to anti‐IgE, despite the strongly diminished expression of FcεRI on basophils by omalizumab. The low pre‐treatment level of degranulation of the basophils might point towards a refractory state of basophils due to activation in CSU. The increase in degranulation was seen in both optimal and suboptimal concentrations of anti‐IgE.

Simultaneously, we found that C5a‐induced degranulation of the basophils was slightly decreased due to omalizumab treatment. This indicates that the observed increase in anti‐IgE–induced degranulation was not an overall increase in intrinsic basophil sensitivity but was specific for the FcεRI‐selective activation routes. This can most probably be explained by the different stimulus‐secretion pathways that are used by FcεRI vs G‐protein–coupled C5a receptors.^33^, ^34^ Our findings are in line with a recently described study by MacGlashan and Saini on cat allergic individuals treated with omalizumab.^35^ This study described that an increased intrinsic basophil sensitivity was the underlying cause of increased IgE‐mediated degranulation of basophils, which was later suggested to be the result of an omalizumab‐induced increased expression of Syk in basophils.^36^ Why this selective responsiveness of basophils changes after omalizumab treatment is unclear; however, it does emphasize the important role of basophils in the mechanism of CSU. We speculate that it might be a reflection of the different maturation state of basophils due to decreased tissue inflammation, which in turn reduces the number of basophils in the skin and potentially leads to a lesser amount of basophil differentiation in the bone marrow.

Neither anti‐IgE– nor C5a‐induced basophil activation was related to treatment response. However, increase in anti‐IgE–mediated basophil activation was most apparent in samples from patients not responding to omalizumab (Figure S1). None of the other cellular responses showed a significant difference between responders and non‐responders; therefore, we were not able to elucidate their role in the omalizumab treatment. These findings imply that the omalizumab‐induced basophil changes might be responsible for the underlying clinical effects, but a yet unknown additional cellular effect could also play a role towards the favourable clinical response.

Notably, both the decrease in FcεRI expression on the cell surface of basophils, and the increase in anti‐IgE–mediated basophil stimulation and decrease in C5a‐mediated basophil stimulation continued for at least 3 months after discontinuation of omalizumab. Given the relative short lifespan of basophils, this suggests either a prolonged effect of omalizumab or involvement of a more complex (possibly intracellular) mechanism of action of omalizumab.

Omalizumab induced a rapid and sustained decline of FcεRI expression on the surface of basophils, pDCs, and mDC CD1c. Despite the diminished expression of FcεRI on basophils by omalizumab, basophil degranulation was significantly increased after the cross‐linking of FcεRI to anti‐IgE. However, none of the findings could predict the response of omalizumab treatment, and more research is therefore required. Our findings suggest that response to omalizumab in CSU patients may be partly explained by pathways involving a high‐affinity IgE receptor of the basophils.

## CONFLICT OF INTEREST

M. van den Elzen received reimbursements to attend symposia and speaker's fees from Novartis Pharma B.V. to the institution. AC Knulst received funds for research and healthcare innovation from Novartis Pharma B.V. to the institution and was involved in the advisory board of Novartis Pharma B.V. H. Röckmann received speaker's fees, and funds for research from Novartis Pharma B.V. to the institution. Remaining authors None declared.

## Data Availability

The data that support the findings of this study are available from the corresponding author upon reasonable request.
