# Supplementary figures and images for: Response of FcεRI‐bearing leucocytes to omalizumab in chronic spontaneous urticaria
Source: Clin Exp Allergy. 2020 Feb 7;50(3):364–71. doi: 10.1111/cea.13566 (PMC7065003; doi:10.1111/cea.13566)

Supplemental figure 1

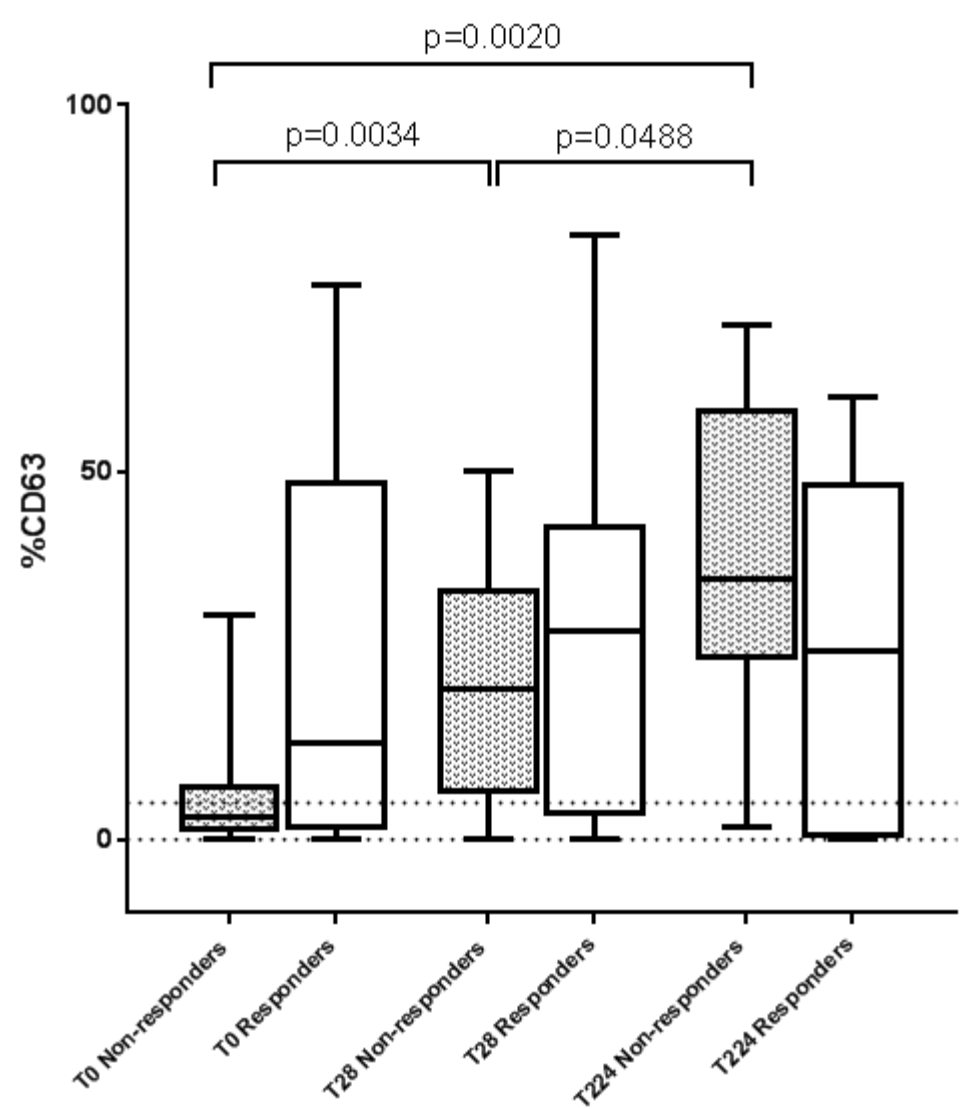

Supplement: Supplementary file 1 [file CEA-50-364-s001.pdf]
