# Supplementary material for: Response of FcεRI‐bearing leucocytes to omalizumab in chronic spontaneous urticaria
Source: Clin Exp Allergy. 2020 Feb 7;50(3):364–71. doi: 10.1111/cea.13566 (PMC7065003; doi:10.1111/cea.13566)
Supplement: Supplementary file 2 [file CEA-50-364-s002.docx]

**Tables**

| Characteristic | Frequency or range |
| --- | --- |
| Median age in years (range) | 42.0 (21 - 70) |
| Female, n (%) | 22 (73) |
| Weight in median kg (range) | 83.7 (61.4 – 114.2) |
| Body mass index median kg/m^2^ (range) | 27.2 (21.2 – 44.6) |
| Presence of angioedema, n (%) | 24 (80) |
| Presence of CINDU in addition to CSU, n (%)  Delayed pressure urticaria  Urticaria factitia | 12 (40)  8 (27)  6 (20) |
| Family history of wheals or angioedema, n (%) | 7 (24) |
| Median disease duration in years (range) , n (%) | 2.7 (0.6 - 29) |
| Atopy by history, n (%)  Any atopy  Atopic dermatitis  Asthma  Allergic rhinitis  Other allergy | 17 (57)  10 (33)  5 (17)  9 (30)  8 (27) |
| Medication use on day of OMA1, n (%)  Second generation antihistamines (sgAH)  First generation antihistamines (fgAH)  H2-antagonist  LTRA | 30 (100)  3 (10)^†^  0 (0)  6 (20) |
| H1-antihistamine dose on day of OMA1, n (%)  Threefold  Fourfold | 2 (7)  28 (93) |
| Previous switch of type of sgAH, n (%) | 17 (57) |
| Previous use of systemic steroids, n (%) | 14 (47) |
| Previous use of immunosuppressants, n (%) | 6 (20) |
| Number of subjects with UAS7 ≤ 6 after one month  Number of subjects with UAS7 ≤ 6 after six months | 6/30 (20)  15/30 (50) |
